# Protocol to identify regulatory modules in Parkinson’s disease progression using miRNA data and Boolean modeling

**DOI:** 10.1016/j.xpro.2025.103769

**Published:** 2025-05-09

**Authors:** Ahmed Abdelmonem Hemedan, Venkata Satagopam, Reinhard Schneider, Marek Ostaszewski

**Affiliations:** 1 Bioinformatics Core Unit, Luxembourg Centre for Systems Biomedicine, University of Luxembourg, 4367 Esch-sur-Alzette, Luxembourg

**Keywords:** Bioinformatics, Molecular Biology, Neuroscience, Systems biology

## Abstract

Regulatory modules are molecules that interact functionally, driving disease processes. Here, we present a protocol for identifying regulatory modules in Parkinson’s disease (PD) using cohort-specific microRNA (miRNA) data and Boolean modeling. We describe steps for omics data collection, biomolecule and miRNA target analysis, and Boolean model construction and simulation. We then detail procedures for validation of the model and results. The modules identified using this protocol explain how miRNA-driven mechanisms influence PD progression in disease cohorts.

For complete details on the use and execution of this protocol, please refer to Hemedan et al.^1^

## Highlights

• Steps for identifying cohort-specific PD modules via Boolean modeling
• Instructions for miRNA target enrichment in PD pathways
• Procedures for simulating molecular shifts in PD cohorts
• Guidance on model validation with experimental PD data

## Abstract (editor highlights)

Publisher’s note: Undertaking any experimental protocol requires adherence to local institutional guidelines for laboratory safety and ethics.

## Graphical abstract

**Figure:**
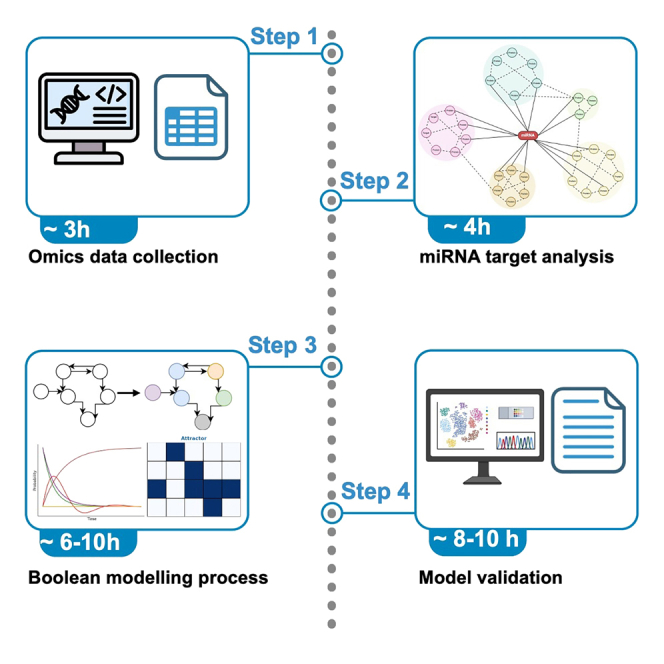

## Before you begin

### Preparation 1: Data access and initial setup

**Timing: 2 h**

1. Download the Parkinson’s Progression Markers Initiative (PPMI) Dataset^2^:
  a. Access the PPMI database through the Laboratory of Neuro Imaging (LONI) archive at www.ppmi-info.org.
  b. Ensure you have an approved account and have signed the data usage agreement.
  c. Download the miRNA expression profiles of blood-derived samples from the following cohorts:
    i. Clinical PD.
    ii. Prodromal PD.
    iii. SWEDD (Scans Without Evidence of Dopaminergic Deficit).

**CRITICAL:** Ensure proper preprocessing of the dataset. Verify that all samples are appropriately annotated and free of missing or inconsistent data, as these issues can introduce bias or errors into downstream analyses.

2. Verify the integrity of the dataset:
  a. Extract the downloaded files and check for completeness.

### Preparation 2: Setting up the software environment

**Timing: 1 h**

3. Install R and DESeq2: Install R (version ≥4.1.0) using your system’s package manager.
  a. For Linux:
    i. With root access.
      sudo apt update
      sudo apt install r-base
    ii. Without root access (using Conda or Mamba).
      conda create -n r_env r-base=4.1.0
      conda activate r_env
  b. For Windows:
    i. Download the R installer from the official CRAN website: https://cran.r-project.org/.
    ii. Run the installer and follow the on-screen instructions to complete the installation.
  c. For macOS:
    i. Download the appropriate R binary for macOS from https://cran.r-project.org/.
    ii. Double-click the downloaded file and follow the prompts to install R.
          Open R and install the DESeq2 package:
      install.packages(“DESeq2”)
4. Install Python and pyMaBoSS^3^: Ensure Python (version ≥3.8) is installed.
  a. For Linux:
    i. Use your system’s package manager to install Python:
      sudo apt update
      sudo apt install python
    ii. Recommended: Using Conda or Mamba (for isolated environments and better package management:
      conda create -n my_python_env python=3.8
      conda activate my_python_env
    iii. Recommended: Using Mamba:
      mamba create -n my_python_env python=3.8
      mamba activate my_python_env
  b. For Windows:
    i. Download the Python installer from the official Python website: https://www.python.org/.
    ii. Run the installer, ensuring you check the box to “Add Python to PATH” during installation.
    iii. For macOS: install Python 3.8 using Homebrew, then use Conda or Mamba to create an isolated environment for better package managment.
  c. Install Python using the official installer from https://www.python.org/.
  d. Double-click the downloaded file and follow the prompts to install Python.
    i. Install the pyMaBoSS package via pip:
      pip install pyMaBoSS
    ii. Ensure pyMaBoSS version 2.0 or higher is installed:
      python -m maboss --version
5. Download and access additional tools:
  a. Download CellDesigner^4^ from celldesigner.org for pathway editing.
  b. Access the Molecular Interaction NEtwoRks VisuAlization (MINERVA) Platform.^5^
    **CRITICAL:** Ensure that the following software versions are compatible to prevent conflicts during analysis:
    i. R and DESeq2: (a) R: Version ≥4.1.0; (b) DESeq2: Latest stable release (compatible with the installed R version). Refer to the Bioconductor documentation to ensure compatibility.
    ii. Python and pyMaBoSS: (a) Python: Version ≥3.8; (b) pyMaBoSS: Version 2.0 or higher (refer to the official repository for updates).
    iii. CellDesigner: Ensure CellDesigner version 4.x compatible with SBML qual format is installed. Check the official website for details.
    iv. MINERVA Platform: Use the MINERVA platform plugin for pathway enrichment and visualization, ensuring compatibility with the uploaded SBML files.
    v. CaSQ (CellDesigner as SBML-qual): Use the version specified in the protocol to ensure successful pathway conversion to SBML qual format (e.g., CaSQ 1.0.3 or higher).

### Preparation 3: Preprocessing miRNA data

**Timing: 3–4 h**

6. Load required libraries: Before running any analysis, load the required R libraries. Ensure that DESeq2 is installed using the command BiocManager::install("DESeq2") if it is not already available.

# Load required libraries

library (DESeq2) # For differential expression analysis

library(readr) # For reading CSV files

7. Load the miRNA expression dataset: Use the following R code to load the miRNA count matrix and sample metadata. Replace the file paths with the actual locations of your datasets (Tables 1 and 2).

**Table 1:** Example format of the miRNA expression matrix

miRNA_ID	sample_1	sample_2	sample_3	sample_4
miR-1	100	150	200	180
miR-2	50	65	80	75
miR-3	300	280	250	270

This table illustrates the expected structure of the input miRNA expression dataset. Rows correspond to miRNAs (identified by miRNA_ID), while columns represent samples with raw read counts.

**Table 2:** Example format of the sample metadata

sample_ID	Condition	Sex
sample_1	control	male
sample_2	disease	female
sample_3	control	female
sample_4	disease	male

This table represents sample metadata that corresponds to the miRNA expression matrix. Each row represents an individual sample, while columns specify sample attributes, including condition (control vs. disease) and sex.

# Load the raw miRNA expression data

countData <- read.csv ("path/to/data.csv", row.names = 1) # Replace with your count data file path (Table1)

# Load sample metadata

colData <- read.csv("path/to/metadata.csv", row.names = 1) # Set sample_ID as row names # Replace with your metadata file path (Table2)

***Note:*** Rows represent miRNAs identified by miRNA_ID. Columns correspond to different samples, with raw read counts as values. The dataset should be formatted as a CSV file, with miRNA IDs in the first column and sample counts in the remaining columns.

***Note:*** The sample_ID matches the column names in countData. The condition indicates sample grouping (e.g., Control vs. Disease). The sex shows females or males. Additional factors can be included, such as age or disease stage).

8. Create a DESeqDataSet object: Organize the miRNA data into a DESeqDataSet object, which is required for downstream differential expression analysis.

# Create a DESeqDataSet object

dds <- DESeqDataSetFromMatrix(countData, colData, design = ∼ condition)

9. Normalize Raw Counts Using DESeq2: Normalize the raw counts to account for differences in sequencing depth and other biases.

# Normalize raw counts using DESeq2

dds <- DESeq(dds)

# Optional: Save normalized counts for inspection normalized_counts <- counts(dds, normalized = TRUE)

# Save normalized data

write.csv(normalized_counts, file = "normalized_counts.csv")

10. Filter differentially expressed miRNAs: Identify differentially expressed miRNAs by applying thresholds for adjusted p-value and log2 fold change.

# Perform differential expression analysis

res <- results(dds)

# Filter for significant miRNAs (adjusted p-value < 0.05 and |log2FoldChange| > 1.5)

res <- res[res$padj < 0.05 & abs(res$log2FoldChange) > 1.5, ]

# Save the filtered list

write.csv(as.data.frame(res), file = "filtered_miRNAs.csv")

**CRITICAL:** Ensure that the filtering thresholds (adjusted p-value < 0.05, log2FC ±1.5) are applied consistently to maintain reproducibility. This threshold ensures that only miRNAs with substantial differential expression are considered for downstream analysis. A log2 fold change greater than 1.5 indicates significant upregulation, while a log2 fold change less than −1.5 indicates significant downregulation. These values were chosen based on established literature and their biological relevance in distinguishing meaningful expression changes in Parkinson’s disease cohorts.

### Preparation 4: Pathway enrichment and model preparation

**Timing: 2–3 h**

11. Perform pathway enrichment.
  a. Connect to the **MINERVA platform** using the minervar package.
    i. Use a public project, such as the Parkinson’s Disease Map.
  b. Retrieve map components and annotation types to identify potential pathways for enrichment.
    # Load the required library
    library(minervar)
    # Define MINERVA API URL and project ID
    map_api <- "https://pdmap.uni.lu/minerva/api/"
    project_id <- "pd_map_spring_24" # Parkinson's Disease Map
    # Retrieve map components
    map_components <- get_map_components(map_api = map_api, project_id = project_id)
    print("Map components retrieved successfully.")
    # Get available annotation types
    annotation_types <- get_annotation_types(map_components)
    print(annotation_types)
  c. Perform pathway enrichment using a predefined list of identifiers (e.g., HGNC_SYMBOL, UNIPROT, ENTREZ).
    # Perform pathway enrichment
    identifiers <- c("PARK2", "LRRK2") # Example identifiers for testing
    enrichment_results <- get_pathway_enrichment( identifiers = identifiers, map_components = map_components, annotation_type = "HGNC_SYMBOL"
    # Adjust based on the annotation types retrieved earlier )
    if (nrow(enrichment_results) > 0) { print("Pathway enrichment results:") print(enrichment_results)
    # Save results to a file
    write.csv(enrichment_results, file = "pathway_enrichment_results.csv")
    } else {
    print("No enrichment results found.")
    }
  d. Retrieve pathway file and visualization.
    # Set the path to the source file
    file_path <- system.file("extdata", "core.xml", package = "minervar")
    # Check if the file exists and proceed
    if (file.exists(file_path)) {
     # Convert the sample file to an image
     converted_image <- convert_to_image(
      source_file_path = file_path,
      source_format = "CellDesigner_SBML",
      target_format = "png"
     )
     # Save the image if conversion was successful
     if (!is.null(converted_image)) {
      output_file <- "pathway_visualization.png"
      writeBin(converted_image, output_file)
      browseURL(output_file)
     } else {
      stop("Image conversion failed.")
     }
    } else {
     stop("Sample file does not exist.")
    }
    **CRITICAL:** Ensure that the correct annotation type is selected based on the available options (e.g., HGNC_SYMBOL, UNIPROT, ENTREZ).

## Key resources table

**Table:** 

REAGENT or RESOURCE	SOURCE	IDENTIFIER
**Software and algorithms**		

pyMaBoSS Framework	GitHub	https://github.com/colomoto/pyMaBoSS
CaSQ Tool for SBML conversion	CaSQ (CellDesigner as SBML-qual)	https://sysbio.curie.fr/projects/casq/
MINERVA Platform (PD map)	MINERVA Platform	https://minerva.uni.lu/
DESeq2 R Package	Bioconductor	https://bioconductor.org/packages/release/bioc/html/DESeq2.html
GSEA Plugin for enrichment analysis	MINERVA GSEA Plugin	https://minerva.uni.lu
CellDesigner (SBML formats)	CellDesigner	https://www.celldesigner.org
Boolean Modeling Framework	GitLab	https://gitlab.lcsb.uni.lu/lcsb-biocore/publications/hemedan23-boolean-modelling-of-pd

**Other**		

Parkinson’s Progression Markers Initiative - miRNA dataset	Laboratory of Neuro Imaging (LONI) archive	https://www.ppmi-info.org/data
PD Map	Luxembourg Centre for Systems Biomedicine	https://pdmap.uni.lu/
Supplemental data	Mendeley data	https://doi.org/10.17632/fs8jfjnxsb.1

## Step-by-step method details

### Data collection and preprocessing

**Timing: 3 h**

This section focuses on preprocessing and normalizing miRNA expression data, followed by calculating effect sizes to identify differentially expressed miRNAs.

1. Download miRNA expression profiles from the PPMI database, ensuring inclusion of clinical metadata (e.g., sex, age, disease stage).
  a. Verify data integrity and consistency across cohorts.
  b. Check for missing values, inconsistencies, or outliers in the miRNA expression data and clinical metadata.
  c. Ensure that all samples have complete and consistent metadata fields (e.g., sex, age, disease stage).
  d. Compare distributions of key variables between cohorts to confirm similarity and correct any discrepancies, such as batch effects or sequencing biases.
  e. Record all steps taken to ensure data integrity.
2. Preprocess and normalize the miRNA expression data.
  a. Use standard normalization techniques to remove batch effects and adjust for sequencing depth.
  b. Apply a log2 transformation to stabilize variance and ensure comparability between samples.
3. Calculate effect sizes for each miRNA between cohorts.
  a. Use Cohen’s distance to measure the magnitude of difference between cohorts (e.g., prodromal vs. clinical PD patients).
  b. Calculate the Common Language Effect Size (CLES)^1^^,^^6^ to estimate the probability that a randomly chosen miRNA expression value from one cohort is higher than that from another cohort.
4. Save the preprocessed data and calculated effect sizes for subsequent analysis.

**Note:** CLES involves comparing every pair of values from the two cohorts to quantify this probability.

### miRNA target identification and pathway enrichment

**Timing: 4 h**

This section focuses on identifying differentially expressed miRNA targets and enriching them to reveal key pathways involved in Parkinson’s disease.

5. Identify miRNA targets using established databases.
  a. Perform a target search across five databases: miRTarBase, TargetScan, DIANA-TarBase, miRDB, and miRWalk.^7^^,^^8^^,^^9^^,^^10^
  b. Extract common targets identified consistently across all the databases to ensure confidence in target selection.
6. Perform pathway enrichment analysis using the MINERVA platform.
  a. Upload the list of identified miRNA targets for pathway analysis.
  b. Focus on pathways associated with Parkinson’s disease, including dopamine signaling, mitochondrial dysfunction, and neuroinflammation.
  c. Extract the most significantly enriched pathways for further modeling.

**Pause Point:** Save the list of enriched pathways and associated target molecules for use in the next step.

### Constructing Boolean models

**Timing: 5–6 h**

**Description**: Construct Boolean models from the identified pathways exported from the previous step.

7. Convert the identified pathways into SBML qual format using the CaSQ tool,^11^ ensuring compatibility with tools like MaBoSS^3^ and GINsim.^12^
8. Simulate the general Boolean models.
  a. Use MaBoSS^3^ to run simulations and observe how pathways behave under different known conditions.
  b. Validate the simulation results by comparing them to experimental data and literature evidence.

### Boolean model calibration and simulation analysis

**Timing: 6–8 h**

**Description**: Fine-tune the Boolean models to reflect cohort-specific parameters and perform simulations to analyze regulatory dynamics.

9. Parameterize the Boolean models using cohort-specific omics data analyzed in Step 1.
  a. Determine the regulatory direction of miRNAs based on their expression levels.
  b. Use the CLES values to assign probabilities of a miRNA regulating its target.

***Note:*** Higher miRNA expression leads to upregulation and stronger target inhibition, while lower expression causes downregulation and reduced inhibition.

10. Calibrate the initial conditions for Boolean models based on the probabilities calculated from miRNA targets.
  a. Set the initial states of each target molecule using probabilities derived from miRNA expression data.
  b. Check the correctness of the Boolean functions to ensure they align with the identified pathways.
11. Perform simulations and analyze outcomes.
  a. Use simulation tools like GINsim^12^ and BoolNet to explore steady states and dynamic behaviors of key pathways.
  b. Identify regulatory modules as groups of molecules whose activity levels and interactions vary significantly between cohorts.
    ***Note:*** These modules represent distinct regulatory structures influencing disease progression.
  c. Analyze how these modules behave in different disease cohorts by comparing their attractor states, stable configurations that define persistent regulatory behaviors under given conditions.
12. Conduct sensitivity analysis to ensure robustness of the models.
  a. Assess the dysregulation effect of each molecule on the molecular dynamic of the identified pathways. To do so, use Rmut package to perform sensitivity analysis against perturbations such as knockout and overexpression.
  b. Use techniques like Monte Carlo simulations used in MaBoSS to estimate the confidence of model predictions.

### Interpretation of results and biological validation

**Timing: 4–5 h**

This section focuses on interpreting simulation results, identifying key regulatory nodes, and validating findings with experimental data and literature evidence.

13. Interpret the outputs of the Boolean models to extract key regulatory modules.
  a. Identify molecular groups forming stable **attractor states**, representing distinct regulatory configurations.
  b. Examine how these modules vary between disease cohorts, capturing differences in molecular activity and regulatory influence.
  c. Investigate the role of miRNA regulation in shaping these modules, highlighting key interactions that shift between PD and control groups.
14. Focus on pathways specific to PD progression.
  a. Determine which pathways are enriched within each identified regulatory module.
  b. Assess how regulatory modules influence disease-related processes such as dopamine metabolism, mitochondrial dysfunction, and neuroinflammation.
15. Compare model predictions with external datasets, such as transcriptomics and proteomics data.
  a. Validate regulatory module behavior by mapping Boolean attractors to experimental expression profiles.
  b. Identify cohort-specific differences in **key pathway activities**, ensuring alignment with known PD mechanisms.
  c. Correlate simulated module activity with clinical outcomes to establish potential biomarkers or therapeutic targets

### Generating visual outputs and reporting results

**Timing: 3–4 h**

This section focuses on visualizing results and preparing a report summarizing the study.

16. Create visual representations of simulation results by overlay the attractor back to the PD map
  ***Note:*** To visualize Boolean simulation results within the PD pathway in MINERVA, we use the Overlays module, which allows users to color molecules based on attractor states.
  a. Prepare a table with attractor states (Table 3).

**Table 3:** Example of attractor states for key molecules

Molecule_name	Attractor_state
molecule1	1
molecule2	0
molecule3	1

This table presents Boolean attractor states for selected molecules in the model.
  b. Upload the file in MINERVA:
    i. Navigate to the overlays section and click overlay.
    ii. Select Upload file and provide the CSV dataset.
17. Create the simulation graph to represent the activity levels of simulated elements over the simulation time in Table 4. To visualize how molecular activity evolves over time, we generate simulation graphs using ggplot2.

**Table 4:** Example of molecular activity over simulation steps

iteration_steps	molecule1	molecule2	molecule3
0	0.2	0.1	0.3
10	0.5	0.2	0.4
20	0.7	0.3	0.6

This table presents simulated activity levels of selected molecules at different time points.

library(ggplot2)

plotSimGraph <- function(file, molecules, output_dir = "simulation_outputs", save_plot = TRUE) {

 data <- read.csv(file, header = TRUE)

 p <- ggplot(data, aes(x = iteration_steps)) +

  geom_line(aes(y = data[[molecules[1]]], color = molecules[1]), linewidth = 1) +

  geom_line(aes(y = data[[molecules[2]]], color = molecules[2]), linewidth = 1) +

  geom_line(aes(y = data[[molecules[3]]], color = molecules[3]), linewidth = 1) +

  theme_minimal() +

  labs(title = "Simulation of Molecular Activity", x = "Simulation Steps", y = "Activity Levels")

 if (save_plot) {

  ggsave(file.path(output_dir, "simulation_plot.png"), plot = p, width = 8, height = 5, dpi = 300)

 }

 print(p)

}

# Example usage

plotSimGraph("simulation_results.csv", c("molecule1", "molecule2", "molecule3"))

To ensure clarity in executing the previous steps, we provide a structured summary of key computational parameters used at each step (Table 5). These parameters define critical thresholds, statistical methods, and modeling configurations necessary for reproducibility. The table below outlines the essential parameters, specifying their corresponding tools and default or recommended values. This structured format allows users to quickly reference settings relevant to their analysis and adjust them based on specific dataset characteristics or experimental needs.

**Table 5:** Key computational parameters used in the protocol

Step	Key parameter	Tool	Default setting & range
**Step 1**: Data Preprocessing	Normalization Method	DESeq2	Median-based (Default), TPM/RPKM (Alternative)
	Differential Expression Cutoff	DESeq2	log2FC ±1.5, *p*-value < 0.05
	Effect Size Calculation	CLES, Cohen’s d	Small (0.2), Moderate (0.5), Large (0.8)
**Step 2**: miRNA Target & Pathway Enrichment	miRNA Target Identification	miRTarBase, TargetScan	High-confidence targets from ≥3 databases
	Enrichment Test & Identifier	MINERVA	Hypergeometric test, HGNC_SYMBOL
	Pathway Enrichment Cutoff	MINERVA	*p*-value < 0.05
**Step 3**: Boolean Model Construction	Model Format	CaSQ	SBML-qual
**Step 4**: Boolean Simulation & Sensitivity	Simulation Iterations	MaBoSS	10,000 (Default), 5,000-50,000
	Sensitivity Analysis	Monte Carlo, Rmut	Knockout, Overexpression
	Attractor Detection	GINsim, BoolNet	Automatic (Default), Fixed-state constraints
**Step 5**: Validation & Interpretation	Boolean Model Validation	MINERVA, Transcriptomics	Overlay attractors with PD pathways

This table summarizes essential parameters applied at each step of the protocol, including normalization methods, statistical thresholds, model configurations, and validation approaches. These settings ensure reproducibility and guide users in adjusting parameters based on their dataset characteristics and experimental requirements.

## Expected outcomes

The expected outcomes of this protocol are focused on identifying critical regulatory pathways involved in PD progression using miRNA targets and Boolean modeling. By leveraging pathway enrichment and cohort-specific simulations, this protocol aims to provide a computational framework for uncovering key disease mechanisms.^6^

This protocol will generate lists of miRNAs that are differentially expressed in PD subgroups compared to the control. Researchers can expect to identify miRNAs with significant effect sizes that influence downstream pathways critical to Parkinson’s disease. The calculation of Cohen’s distance and Common Language Effect Size (CLES) will enable the identification of miRNAs with the most substantial regulatory impact.

Researchers will identify miRNA targets and extract high-confidence ones. The enrichment of these targets using the MINERVA platform is expected to highlight critical pathways, such as those involved in dopamine synthesis, mitochondrial function, and neuroinflammation. The enriched pathways will be converted to Boolean models, helping to simulate and understand how dysregulation of miRNAs and targets influence the pathways. Researchers can expect to generate SBML qual models compatible with simulation tools and platforms (e.g., MaBoSS and CellCollective).

The protocol includes the use of CLES to translate miRNA expression levels into probabilistic assessments of target molecule states. Researchers can expect to derive probabilistic values for whether a target is upregulated or downregulated, considering miRNAs’ inhibitory roles. For targets regulated by multiple miRNAs, the combined probability calculations will enable a comprehensive understanding of how these miRNAs interact to influence disease-related pathways.

Finally, running simulations with the generated Boolean models will allow researchers to explore pathway dynamics under different scenarios, helping to identify critical regulatory nodes and potential points of intervention.

## Limitations

This protocol offers an approach to understanding the role of miRNAs in Parkinson’s disease progression. However, the accuracy of the results depends on the quality of the input data; thus, preprocessing and normalization are essential to minimize potential biases.^6^^,^^13^^,^^14^ While leveraging multiple miRNA target databases significantly enhances confidence in target identification, it may also be conservative, possibly overlooking less-characterized or context-specific interactions. To overcome this challenge, we performed extra step, filtering the miRNA and targets that expressed in substantia nigra, specific tissue for PD, and validated in the context of Parkinson’s disease.^6^^,^^15^

The Boolean modeling framework allows identifying key regulatory dynamics, yet it simplifies complex molecular behaviors into binary states. While this helps understanding major regulatory changes, it may not fully capture quantitative variations in expression levels.

A key challenge in this protocol is the integration of multiple platforms (R, Python, CellDesigner, MINERVA, and CaSQ). While each tool has a non-redundant role, users unfamiliar with these tools may require additional time for setup and learning. To facilitate this, the key resources table provides direct links to documentation and relevant examples.

## Troubleshooting

### Problem 1

Inaccurate miRNA target identification due to variability in miRNA expression data across different datasets (related to Step 2).

### Potential solution

• Ensure that the raw data is preprocessed and normalized to account for batch effects, sequencing depth, and sample variability. Consistent normalization protocols can help minimize biases in the expression data.
• When integrating targets from multiple miRNA databases, discrepancies can arise. Focus on identifying common targets across at least three databases to improve the confidence level. Additionally, consider validating key targets using independent experimental data if available.
• For miRNAs with borderline expression changes, modify cutoff thresholds for target inclusion to reduce false positives, and cross-validate with pathway enrichment outcomes to confirm biological relevance.

### Problem 2

Pathway enrichment using the MINERVA platform may result in low pathway coverage for specific miRNAs targets (related to Step 2).

### Potential solution

• Double-check that the miRNA target list includes only high-confidence targets before uploading to the MINERVA platform.
• For miRNAs with limited known targets, consider using broader pathway databases or relaxing pathway inclusion thresholds slightly to capture related pathways.
• If certain critical pathways are consistently missing, manually inspect the pathway interactions and consider adding custom pathways or adjusting the enrichment parameters.

### Problem 3

Performing simulations without checking the model correctness may provide inconsistent results (related to Step 3).

### Potential solution

• Ensure that the initial conditions for the Boolean model are set correctly based on the cohort-specific parameters derived from the miRNA data. Adjust the probabilities to reflect actual miRNA influence more accurately.
• If the model does not converge, experiment with adjusting the Boolean functions for key regulatory nodes or tuning the simulation parameters (e.g., number of iterations, stochastic noise levels).
• Check the integrity of the SBML qual files and confirm that the conversion from CaSQ was performed correctly. Running a test simulation using a simpler dataset can help identify any underlying issues in the model configuration.

### Problem 4

Incorrect or missing output in simulation results due to parameter misconfiguration (related to Step 4).

### Potential solution

• Review the parameter settings for each cohort, ensuring that they are aligned with the biological context of Parkinson’s disease. Misconfigured parameters can lead to misleading results.
• Double-check that cohort-specific Boolean models are being loaded with the correct input files. Running a test simulation with known outputs can help confirm accuracy.
• If using different simulation platforms, confirm compatibility of the SBML Qual format with each platform, particularly when transitioning between MaBoSS and GINsim.

### Problem 5

Difficulty in validating the Boolean model outputs against biological data due to the complexity of PD pathways and the variability in molecular influence across different cohorts (related to Step 5).

### Potential solution

• Integrate publicly available Parkinson’s disease datasets, ensuring alignment with your study’s molecular focus (e.g., miRNA expression or pathway-specific markers) and cohort characteristics (e.g., sex, disease stage). Focus on well-curated datasets that directly support cross-validation of Boolean model outputs by providing complementary molecular evidence. This ensures the findings are both biologically relevant and consistent with established data sources.
• Use sensitivity analysis to highlight the robustness of your model predictions. By systematically varying the expression levels of critical miRNAs, you can identify key regulatory nodes that consistently impact pathway behavior, enhancing confidence in your model’s biological relevance.
• Engage experts in neurobiology or clinical research to interpret the computational findings, ensuring that the simulated results are aligned with known disease mechanisms and can be translated into meaningful biological insights.

## Resource availability

### Lead contact

Further information and requests for resources and reagents related to this protocol can be directed to Dr. Ahmed Abdelmonem Hemedan (ahmed.hemedan@uni.lu). All inquiries will be handled by the lead contact.

### Technical contact

For technical questions about executing this protocol, Dr. Ahmed Abdelmonem Hemedan (ahmed.hemedan@uni.lu) will provide detailed guidance and support to ensure correct implementation.

### Materials availability

This protocol primarily utilizes publicly accessible computational tools, datasets, and resources. No new biological or chemical reagents were generated during this study. Key datasets, such as miRNA expression profiles from the Parkinson’s Progression Markers Initiative (PPMI), are publicly available. Additionally, enriched pathway information and Boolean models were derived from open-access data and computational resources. Researchers can access the PD Map and MINERVA platform to reproduce the analyses performed in this study.

### Data and code availability

All datasets analyzed in this protocol are available from publicly accessible repositories:

• PPMI miRNA dataset: https://www.ppmi-info.org/data.
• PD Map: https://pdmap.uni.lu.
• Pathway enrichment results and Boolean models: MINERVA Platform.

All computational scripts, including those for miRNA target identification, pathway enrichment, and Boolean model simulations, are available in the GitLab repository: https://gitlab.lcsb.uni.lu/lcsb-biocore/publications/hemedan23-boolean-modelling-of-pd. You can cite all versions by using the zenodo https://doi.org/10.5281/zenodo.15020094. Original data have been deposited to Mendeley Data: [doi.org/10.176232/fs8ifjnxsb.1]

For additional details, visit the following GitHub repositories related to the main tools used in this study:

• pyMaBoSS framework: https://github.com/colomoto/pyMaBoSS.
• CASQ Tool for SBML conversion: https://casq.readthedocs.io/en/stable/.

All scripts, models, and results have been made publicly available to support reproducibility and transparency in scientific research.

## Acknowledgments

This research was funded by the European Union’s Horizon 2020 program, under grant agreement no. 733100: SYSCID—A systems medicine approach to chronic inflammatory diseases. The authors extend their gratitude to the Parkinson’s Progression Markers Initiative (PPMI) for providing access to the data used in this study. PPMI is supported by the Michael J. Fox Foundation for Parkinson’s Research.

## Author contributions

A.A.H.: investigation, conceptualization, and writing, editing. V.S.: supervision, and contributed to reviewing and editing. R.S.: supervision and engaged in reviewing and editing. M.O.: conceptualization, review, editing, and overall supervision. All authors reviewed and approved the final version of the manuscript.

## Declaration of interests

The authors declare no competing interests.
